# A structured approach to a diagnostic of collective practices

**DOI:** 10.3389/fpsyg.2014.01418

**Published:** 2014-12-05

**Authors:** Cristina Bicchieri, Jan W. Lindemans, Ting Jiang

**Affiliations:** ^1^Behavioral Ethics Lab, Philosophy, Politics and Economics, University of PennsylvaniaPhiladelphia, PA, USA; ^2^Behavioral Ethics Lab, Department of Philosophy and Psychology, University of PennsylvaniaPhiladelphia, PA, USA

**Keywords:** social norms, child marriage, monitoring and evaluation, surveys, experiments, vignettes

## Abstract

“How social norms change” is not only a theoretical question but also an empirical one. Many organizations have implemented programs to abandon harmful social norms. These programs are standardly monitored and evaluated with a set of empirical tools. While monitoring and evaluation (M&E) of changes in objective outcomes and behaviors is well-developed, we will argue that M&E of changes in the wide range of beliefs and preferences important to social norms is still problematic. In this paper, we first present a theoretical framework and then show how it should guide social norms measurement. As a case study, we focus on the harmful practice of child marriage. We show how an operational theory of social norms can guide the design of surveys, experiments, and vignettes. We use examples from existing research to illustrate how to study social norms change.

## INTRODUCTION

“How social norms change” is not only a theoretical question but also an empirical and a practical one. Many organizations—governmental, intergovernmental, and non-governmental—try to design programs to end harmful social norms that impact the well-being of millions of people. These programs are standardly monitored and evaluated with a set of empirical tools. While monitoring and evaluation (M&E) of changes in objective outcomes and behaviors works well, we will argue that M&E of changes in the wide range of beliefs and preferences important to social norms is still problematic. Social norms are a kind of collective practice. Measurement should be guided by an adequate theory of collective practices that can be operationalized in order to distinguish social norms from other practices. Such a theory is often lacking. In this paper, we present a structured approach to the diagnostic of collective practices. We first introduce a theoretical framework and then show how it can guide measurement.

To illustrate how to add social norms to an M&E framework, we focus on the harmful practice of child marriage^1^, as it provides an interesting case study. Child marriage is on the decline, but it is still a widespread practice. It occurs in many different areas of the world, from Africa to the Asian subcontinent. It is a violation of human rights—more specifically, the United Nations Convention on the Rights of the Child—and it has very harmful consequences: (1) even when the marriage is not forced, young girls are often too immature to give their well-reasoned consent to the marriage (Mikhail, 2002; Gaffney-Rhys, 2011;); (2) by taking away childhood and the chance of an education, child marriage inhibits girls’ personal development and makes learning and practicing a profession extremely difficult (Amin et al., 1998; Mikhail, 2002); (3) girls risk sexual abuse and violence by their husbands (Raj et al., 2010); and (4) early pregnancies increase risks of disease and even death of mother or child (Mahavarkar et al., 2008; Raj, 2010; Raj and Boehmer, 2013).

There has been much research on child marriage, both empirical and theoretical, with an eye to abandoning it. Scholars have developed tools to measure different aspects of child marriage and they have offered explanations about the origin and persistence of this practice. While there has been substantial progress in child marriage research, we believe that the existing research suffers from three problems. First, there are too many explanations of child marriage, and it is not clear how the different determinants identified by these explanations fit together. Second, explanations of child marriage often lack theoretical rigor. Some scholars might recognize that child marriage is a “social norm,” but at the same time they might refer to it as “culture,” a “practice,” an “institution,” a “custom,” a “convention,” or a “moral” imperative, and there is no theory behind any of this^2^. Related to this is the third problem: there are measurement tools available for M&E of progress in ending child marriage, including tools to get at “social norms,” but these instruments also lack rigorous theoretical backing.

In this paper, we offer a general theoretical framework that can, first, help integrate the different explanations of child marriage and, second, guide the development of measurement tools essential for child marriage M&E. Our theoretical framework approaches social norms through insights into how individuals make decisions. The collective practice of child marriage is ultimately a cluster of individual behaviors, so that, if we want to understand it, we have to understand why individuals behave in certain ways. One important aspect of people’s behavior is that it is often influenced by what others do and by what others think should be done. A social norm is present when behavior is influenced in that manner (Bicchieri, 2006, 2014). We show that a rigorous theoretical framework based on these ideas is a useful guide for the M&E of progress in changing social norms.

In the second section, we introduce a simple model that explains the behaviors of individuals—including the behaviors that sustain the practice of child marriage—by the *preferences* they have, the *options* they have to choose from, and the *beliefs* they have about these options. We argue that collective practices like child marriage can be sustained by two kinds of preferences, namely *unconditional* and *conditional preferences*, and two kinds of beliefs, namely *non-social beliefs* and *social expectations*. Mapping the full range of preferences and beliefs makes it possible to determine whether a collective practice is a social norm or not. In the third section, we discuss different types of practices (customs, moral rules, conventions, social norms, etc.), and we show why it is crucial, for designing effective interventions, to understand what type of practice child marriage is. We also show that collective practices can be sustained by false beliefs. Because informing people about the falsity of these beliefs can contribute to ending child marriage, we argue that it is important that child marriage M&E includes measuring the “local knowledge” that may generate false factual beliefs as well as the social expectations that may prove to be wrong.

In the next three sections we assess different measurement tools and their role in a *diagnostic for collective practices*. In the fourth section, we discuss the most commonly used tool in research on collective practices, namely “knowledge, attitude, and practice” surveys. We argue that even if one refines these surveys several problems remain, namely, how to evaluate the causal role of beliefs, how to obtain truthful answers, and how to obtain accurate answers. In the fifth section, we show that incentivized experiments offer a partial remedy. Laboratory experiments, however, take place in rarefied conditions and in the sixth section we argue that in-kind incentives and vignettes provide a more realistic solution that mimics experiments while being feasible in the context of harmful practices like child marriage. The last section concludes the paper.

## CHILD MARRIAGE AND THE *PREFERENCES, OPTIONS, AND BELIEFS* MODEL

Monitoring and evaluating progress in ending social norms requires a solid theoretical framework. In this section we present such a framework based on a simple model of decision-making, namely the *preferences, options, and beliefs* model (see Gintis, 2007). To illustrate the necessity of a general theoretical framework, we discuss the status quo in research on understanding child marriage.

Child marriage does not lack explanations. If anything, child marriage has too many explanations. Here is a list of explanations that have been given for the origin and persistence of child marriage (see for instance UNICEF, 2001; Jain and Kurz, 2007; Loaiza and Wong, 2012; Verma et al., 2013):

(1) **Conciliation:** Marriages are primarily an instrument to bring families closer together, pay debts, or solve conflicts.
(2) **Why-Educate:** Parents are too poor to pay for the girl’s upbringing, and marriage means one less mouth to feed. Moreover, there are no schools in the neighborhood, and there are no jobs for women.
(3) **Dowry:** Parents have to pay higher dowries or accept lower bride prices for older girls. Potential grooms, or their families, prefer young brides.
(4) **Safeguard:** If (good) grooms are scarce, it is best to marry whenever a (good) possibility arises.
(5) **Chastity:** Parents want their daughters to be chaste, and there is a risk that girls who grow older lose their virginity outside marriage, because they might have love affairs or they might be raped. Here are some variants of the Chastity explanation:
**         • Ignorance-about-Chastity:** Parents overestimate the risk of love affairs (and rape).
**         • Chastity-Norm:** Daughters are expected to be chaste, and the slightest suggestion of premarital sex would ruin the reputation of both daughter and parents.
**         • Ignorance-about-Chastity-Norm:** Parents overestimate the extent to which others expect them to have chaste daughters.
(6) **Conformity:** All girls are getting married young. Here is a variant of the Conformity explanation:
**         • Ignorance-about-Conformity:** Parents overestimate the number of girls that are getting married young.
(7) **Tradition:** Child marriage is just a “custom,” a “tradition,” part of people’s “culture.”
(8) **Housewife:** People, girls included, believe girls should be good wives and mothers, and their well-being and personal development is less important.
(9) **Ignorance-about-Harm:** People underestimate the harm child marriage causes.
(10) **Docility:** Girls lack the capabilities to promote their own interests.
(11) **Law:** There are no laws forbidding child marriage or, if there are, they are not enforced. Here is a variant of the Law explanation:
**         • Ignorance-about-Law:** The laws forbidding child marriage are not known.
(12) **Juliet:** Children themselves desire love and marriage.

You might wonder: if there are so many explanations already, why do we still need a theoretical framework to guide M&E? The answer is that the listed explanations do not provide a sufficiently general theoretical framework. They are partial explanations rather than encompassing theories of child marriage. The listed explanations explain only some features of child marriage, and they apply only to some regions and to different times. Hence, we still need an encompassing theory to develop a systematic approach to monitoring change in child marriage. This theory needs to be sufficiently general so that it can include all the partial explanations listed above.

To build such a general theoretical framework, we start with a simple economic model of behavior and gradually add refinements. According to economists, people behave in a certain way because they maximally satisfy their *preferences* given the limited *options* they have. The Why-Educate explanation nicely illustrates this economic way of thinking about decision-making. A father prefers to have as much food, clothes, and other goods as possible, but his options are limited. First, he faces a budget constraint: the money he invests in his daughter’s education—in the hope that she will get a job and provide for the family—cannot be used to buy goods today. He will have to choose. Second, there are structural constraints: if there are no schools for girls or no jobs for women, the return on the father’s investment is nil. The Dowry explanation fits the preference-satisfaction-given-limited-options mold too. Like education costs, dowries are costs that limit parents’ options: they cannot both save money on a lower dowry and postpone the wedding—they have to choose. To understand people’s behavior we thus need to understand both people’s preferences and the limited options they have when trying to satisfy these preferences.

Now, the preferences of parents are of course not purely *self-regarding*. Parents also care about their daughters: they want them to find a good husband, to be good wives, to have children, to be happy, etc. They want that for their daughters, not necessarily because they will get something from it. In other words, parents have *other-regarding preferences*: preferences about the well-being of others rather than their own well-being. Other-regarding preferences also play a role in explaining child marriage. According to the Safeguard explanation, if grooms are scarce and parents want their daughters to enjoy the bliss of marriage, they will marry them off when the opportunity arises. This means that parents maximally satisfy other-regarding preferences given the limited marriage options they have. Likewise, if the slightest suggestion of premarital sex would ruin the reputation of a girl and thus her chance of getting married, it is in her own interest to be married off early, and some parents do so for the sake of their daughters—this is a variant of the Chastity-Norm explanation. Since preferences—self-regarding and other-regarding—play an important role in explaining the behavior of the parents, child marriage M&E must carefully elicit these preferences.

Earlier we mentioned that child marriage is detrimental for the girls’ development and health. So you might wonder why parents still marry off their daughters so young if they care about their well-being. According to the Ignorance-about-Harm explanation, parents might just underestimate the extent of the harm child marriage causes. Parents might falsely believe that the benefits of an early marriage outweigh the harm. Hence, to understand how people make decisions, we need to understand not only (1) their *preferences* and (2) the limited *options* they have in trying to satisfy these preferences, but also (3) the (true or false) *beliefs* they hold about their options. This implies that programs to end harmful norms can aim at changing preferences, options, and/or beliefs, and M&E will have to encompass measures of (changes in) preferences, options, and/or beliefs.

We now gradually refine the *preferences, options, and beliefs* model, by clarifying the kinds of preferences and beliefs that influence people’s behavior. The Chastity explanation, for instance, is ambiguous about the type of beliefs that support the child marriage decision. Parents may have prudential reasons to demand chastity: they may believe that, if the girl is not married off early, she might have love affairs and ruin her chance of a decent marriage. However, parents might also have moral reasons: they might believe that women should be chaste and that it would be morally bad for the daughter to have premarital sex. In other words, their belief might not be factual, but *normative*, namely a belief about what should be done—and what should not be done. Normative beliefs often make people punish those who do not do what they should do by gossiping about them, by socially excluding them or worse, as when a girl is killed to restore family honor.

There is yet another alternative. Parents might not believe themselves that girls should be chaste, but they might believe that *others* believe girls should be chaste, and parents do not want to go against the normative beliefs of others—or they do not want to get punished. Beliefs about what *others* think should be done—and whether others might punish deviants—are called *normative expectations* (Bicchieri, 2006). They contrast with personal beliefs about what should be done, which we call *personal normative beliefs*. While personal normative beliefs are first-order beliefs, normative expectations are second-order beliefs: beliefs about what others believe, that is, “beliefs about beliefs” (Bicchieri, 2006, p. 15, 2014, Chap. 1). More specifically, normative expectations are expectations about others’ personal normative beliefs—and about the sanctions they may enforce.

Another important set of beliefs is beliefs about what others *do*, as people seldom care only about what others *think* should be done (Bicchieri and Xiao, 2009). Parents might decide to marry off their young daughters because they believe other parents do so. Beliefs about the behavior of others are called *empirical expectations* (Bicchieri, 2006). Empirical and normative expectations are beliefs about others; we call these beliefs about others *social expectations*.

The personal belief that one should behave in a certain way is not a belief about others—it is not a “social” belief. The same can be said about the beliefs about the consequences of early marriage. We call the latter *factual beliefs*, in contrast with both personal normative beliefs and social expectations. Like personal normative beliefs, factual beliefs are “non-social”; like empirical expectations, factual beliefs are “non-normative.” **Table 1** illustrates the two dimensions according to which we can categorize beliefs: their being social or not, and their being normative or not.

**Table 1:** Classification of beliefs.

	Non-social beliefs	Social beliefs/expectations
Non-normative beliefs	Factual beliefs	Empirical expectations
Normative beliefs	Personal normative beliefs	Normative expectations

## DISTINGUISHING SOCIAL NORMS FROM OTHER COLLECTIVE PRACTICES

The refined *preferences, options, and beliefs* model makes it possible to give a clear definition of what a social norm is and to distinguish social norms from other collective practices. In this section, we explain how to categorize collective practices and why knowing exactly what kind of collective practice we are dealing with is important when we want to enact change.

For identifying social norms, the distinction between non-social beliefs and social expectations is crucial. We call people’s preferences that are conditional on social expectations *conditional preferences*, in contrast with *unconditional preferences* like self-regarding, other-regarding, and moral preferences. The latter are not conditional on what others do or think. A social norm then is a collective practice sustained by empirical and normative expectations and by preferences conditional on both these expectations (Bicchieri, 2006, p. 11).

This definition should be further specified in the following ways. First, not all social expectations matter for social norms. When a father’s preferences about his daughter’s marriage are conditional on his expectations about what others do and think, he does not care about what people do and think in other countries, cities, or far away villages. He will care about what specific people do or think, namely those who belong to his *reference network* (Bicchieri, 2014, Chap. 1). The reference network of the father in our example might include other families in his village, the village elders, religious leaders, and perhaps also relatives in distant villages. Who exactly belongs to people’s reference network is an empirical question. Second, it will seldom be the case that everybody in one’s reference network will behave and think in the same way on every issue. But it is enough that many people behave or think in a similar way for people to be influenced. Exactly how much collective conformity is necessary to influence one’s behavior is again an empirical question, but it is easy to think of social norms as being based on expectations about at least a majority (Bicchieri, 2006, p. 12).

Scholars sometimes conclude too quickly from the fact that a practice is widespread that it must be a “social norm.” However, not all collective practices are social norms, as our discussion of the different explanations of child marriage should have illustrated. By merely observing a collective practice like child marriage, we do not know what is the nature of the practice, because we do not know why people endorse it.

First, as the Why-Education and Dowry explanations illustrate, the practice of child marriage could just be sustained by self-regarding or other-regarding preferences, in which case it would be a *rational response.* For instance, parents might be just calculating that it would cost too much money to keep their daughters at home. If all parents think like that, you may observe a homogenous collective practice that is the result of each individual calculating what best fulfills his or her interest regardless of what others do.

Second, child marriage might be just something that is traditionally done in certain communities. The reason for it might have been long forgotten, but people still do it because that is what they have been taught to do. In that case, we would call child marriage a *custom*.

Third, child marriage could also be sustained by moral preferences, based on personal normative beliefs, which would make it a *moral rule*. If child marriage is mainly due to parents holding the *personal normative belief* that women should be chaste and protected, it is a moral rule. Of course, in a community that shares moral rules, parents may also have (correct) normative expectations about what other parents believe one should do. Yet we should be able to distinguish between the influence of personal normative beliefs and normative expectations. If child marriage were to be primarily due to parents having the *normative expectation* that others hold that women should be chaste and protected, regardless of the personal normative beliefs they may hold, then we would be entitled to say it is a social norm.

Finally, even if child marriage were to depend on social expectations, it is still possible that it is not a social norm, because it is at least theoretically possible that it would depend on empirical expectations alone. Practices that depend solely on empirical expectations are called *descriptive norms* (Bicchieri, 2006, 2014). Driving on the right side of the road is an example of a descriptive norm or, more specifically, a *convention*. Driving on the right side of the road is not a social norm because people’s driving on the right side is not conditional on their normative expectation that others believe they should drive on the right side. Rather, they simply do not want to crash into other people. Child marriage is probably not a convention like driving on the right side of the road, although there are conventional aspects to child marriage: the parents of girls have to coordinate with the parents of boys (or with men) on when to marry. In line with the Chastity explanation, normative expectations most likely matter too.

Knowing whether child marriage is a rational response, a custom, a moral rule, a descriptive norm, or a social norm is very important for designing effective policies. If child marriage is a rational response, the incentives need to be changed. If child marriage is a custom, it is fairly easily abandoned: if people realize that the reason why a certain custom was once established no longer holds, they will be inclined to abandon it, especially when presented with satisfactory alternatives.

Whether child marriage is a descriptive norm or a social norm, change must still occur collectively, in a coordinated way (Mackie, 1996; Bicchieri, 2012; Bicchieri and Mercier, 2014). Since people’s behavior depends on what others in their reference network do and think, they will not change their behavior unless others do so as well. Hence, the entire reference network needs to participate in the change. We cannot adopt a piecemeal approach, starting with a few individuals and hoping to expand gradually.

Moreover, if a practice is a social norm rather than a descriptive norm, it is important to change normative expectations. In the case of child marriage, this might mean that one has to change not only people’s personal normative beliefs that child marriage is valuable but also their normative expectations that others who matter to them value and approve of child marriage.

Finally, whatever kind of practice child marriage is, it is possible that it is based on *false beliefs*, which opens new routes for intervention. Although one cannot argue about preferences, one can challenge beliefs^3^. From an M&E point of view, this means that it is important to measure not only beliefs but also the things that make these beliefs true or false. All sorts of beliefs related to child marriage could be false. People’s factual beliefs may be false. For instance, a father’s belief that an unmarried girl’s integrity and purity are doomed to be damaged may be false. He may also falsely believe that the law permits child marriage, or that the younger the girl, the easier it will be for her to get attached to her husband and his family.

Similarly, people’s social expectations can be false. First, their empirical expectations can be false: a father might falsely believe that almost everybody marries off their daughters young and underestimate the number of later marriages. Second, people’s normative expectations can be false: a father might overestimate the number of people who believe that chastity is important. In the very extreme case, it can be that everybody thinks that others endorse the practice but, in fact, only a minority does. Because the practice is widespread, but people fear to put themselves at a disadvantage by telling what they really think, everybody will publicly endorse it, hence reinforcing a social norm most people dislike. The situation in which a norm persists because many people are ignorant about others’ true beliefs is called *pluralistic ignorance*.

That collective practices, be they norms or shared rational responses, can be based on false beliefs is important for interventions. If any of the factual beliefs people hold is false, the possibility to inform them about the truth is a powerful tool. Similarly, if social expectations are incorrect, making individuals’ true beliefs public is an important step toward change.

## THE USUAL MEASUREMENT TOOL: SURVEYS AND THEIR LIMITS

In the following sections, we make use of the categorization of collective practices we just described to construct a diagnostic tool for determining the exact nature of a collective practice. In this section, we will discuss how to employ traditional surveys to acquire information about people’s behaviors, preferences, and beliefs about their options. In the next section we will discuss what we can learn from behavioral experiments about incentivizing accurate answers, and in Section “More Realistic Tools: In-Kind Incentives and Vignettes” we look at other tools that better suit the needs of organizations looking to evaluate their programs in the field.

A very popular tool to investigate the determinants of collective practices is the knowledge, attitudes, and practices (KAP) survey. KAP surveys are often part of the M&E of programs to end harmful practices, like child marriage (e.g., Save the Children Norway, 2011). Unfortunately, as the name indicates, KAP surveys typically measure only factual beliefs (“Knowledge”), personal normative beliefs (“Attitudes”), and collective behaviors (“Practices”). They typically do not elicit social expectations.

Some surveys, however, do include social-expectations questions. Some large-scale household survey programs, like USAID’s Demographic and Health Surveys (DHS) and UNICEF’s Multiple Indicator Cluster Surveys (MICS), have been refined to incorporate social-norms related questions. Some of the smaller, country-specific surveys, often part of the M&E of programs, also provide data from a social norms perspective. For instance, Maharjan et al. (2012) asked respondents “What are the positive effects of child marriage?,” and Sood et al. (2007) asked “Approximately, how many girls are married early in your community?” and “Are people in your area in favor of child marriage?” The resulting data can give us indications about factual and personal normative beliefs, empirical and normative expectations. Such data are the building blocks in constructing a *diagnostic of child marriage*.

However, even if we piece together questions from several surveys, more questions—and more carefully designed questions—are needed to correctly diagnose the presence of social norms. Because the above surveys were not designed with an adequate theory of norms in mind, none of them clearly distinguish between personal normative beliefs and normative expectations. We argue that a diagnostic of child marriage requires at least the following questions:

• BEHAVIOR: “At what age did your daughter(s) get married?”
• PRUDENTIAL REASONS: “If you think about a girl marrying early rather than late, what are the advantages and disadvantages of that for the father of the girl?”
• EMPIRICAL EXPECTATION: “Think about married women in between 18 and 25 years old in your community. Out of 100 such women, how many do you think got married before they were 18 years old?”
• PERSONAL NORMATIVE BELIEF: “Some girls get married before they are 18 years old. Is this good?”
• NORMATIVE EXPECTATION: “Out of 100 men in your community who are at least 40 years old, how many think that it is good that girls get married before they are 18 years old?”

The diagnostic would work as follows. If fathers have strong prudential reasons for their daughters to marry—like having to pay a smaller dowry—then child marriage could be a *rational choice* or a *custom* followed because it is in fathers’ own interest. If fathers have strong personal normative beliefs, then child marriage could be a *moral rule*. If there are no strong prudential reasons or personal normative beliefs, but if people consistently hold the empirical expectation that most other fathers are marrying their daughters off early, then child marriage is probably a *norm*. If people also hold the normative expectation that others think girls should marry early, then child marriage is probably a *social norm*—otherwise it is probably a *descriptive norm*.

None of these diagnoses are final, however: the above questions separate different kinds of motives, but this does not mean that some of them cannot be present together. One may have prudential and moral reasons as well as social expectations. What matters is to uncover which motives influence behavior, and to what extent—this is what ultimately determines the real nature of the practice. Even if we know people’s prudential reasons, personal normative beliefs and social expectations, we still need to find out what *causal role* these potential motives play. To understand whether social norms have causal influence, we want to *manipulate* expectations and see how individuals’ choices change (or would change). Having conditional preferences means precisely that: were social expectations to change, behavior would change, too. This can be best done with behavioral experiments and vignettes, which we shall discuss in the next two sections.

There is a second, major problem with surveys. In countries where governments criminalize child marriage and NGOs conduct programs trying to end it, child marriage is morally, socially as well as legally a sensitive issue. This difficulty implies that traditional questionnaires might not give us reliable information (Raj et al., 2011, p. 13). People’s expressed evaluation of child marriage might be unreliable because of a *social desirability bias*—a tendency to respond in ways that are thought to be appropriate. Respondents might give morally, socially, or legally “correct” answers rather than answers that reflect their true beliefs. Such bias is especially likely when the research is conducted by organizations with a clear pro-child agenda.

Social pressure might point in different directions. People’s answers might conform to what they think government and the NGOs approve of—marrying late, so as to enable education for the girl. But they might also conform to what they think their community approves of—marrying young, so as to ensure a husband for the girl. One might think that the latter bias is not so problematic for identifying the presence of a norm. However, as we argued earlier, to diagnose pluralistic ignorance, we need to accurately elicit both personal normative beliefs and normative expectations. If expressed personal normative beliefs merely reflect normative expectations, such a diagnosis becomes impossible.

There have been several ways of dealing with the social desirability bias that have been proposed over the years, and some having been proven to be more effective than others. Crowne and Marlowe (1960) proposed that social desirability should be measured as a dispositional trait so that it could be controlled for in analyses. Unfortunately their proposed measurement (and similar alternatives; e.g., Reynolds, 1982) was developed for use in an American population and validated with an American college sample, so many of the behaviors referred to in the survey may not be considered “socially (un)desirable” in other cultures.

Beyond detecting and controlling for social desirability, researchers have proposed ways to actively reduce social desirability in respondents. For example, one method is to force respondents to choose between two or more equally socially desirable options (e.g., Feldman and Corah, 1960). Unfortunately, this particular method relies on the questionnaire designer to guess what choice options would be equally socially desirable to the typical respondent, which is often difficult to infer and may differ from respondent to respondent. Other options include the “bogus pipeline” method that entails telling the participant that they are being monitored by some form of lie detector, thereby signaling to them that dishonesty is futile (Jones and Sigall, 1971; Nederhof, 1985). This particular method is problematic not only because it necessarily entails deceiving a participant (which can reduce their trust in the researcher, especially if respondents see through the deception), but because it requires the researcher to bring a fake machine with them that looks convincingly like a lie detector, something that would be particularly difficult in field settings. Another example of a method of minimizing social desirability would be to maximize the respondents’ anonymity (Nederhof, 1985). Anonymity, however, is difficult to attain when dealing with practices such as child marriage in rural areas where measurements have to be administered in person. Moreover, even if full anonymity is attained, respondents may continue to “lie to themselves” and respond in a socially desirable way.

A more promising method to assess personal normative beliefs (and obtain truthful answers) is the so-called randomized response technique (Greenberg et al., 1969). Respondents secretly throw a coin and must respond “yes” if it comes up tails, and are instructed to respond truthfully if it comes up heads. Since anonymity is guaranteed, it is assumed that those who get heads will tell the truth. For example, in a community that widely practices child marriage, the question should be “Some girls get married before they are 18 years old. Is this good?”: half of the responders get tails, so half of the queried population will answer “yes” regardless of whether they like the practice. Whatever is the proportion of responders who say “no,” the true number is double that amount since it is assumed that in a large randomized sample the two halves are approximately the same. This enables the researcher to estimate the actual prevalence of supporting beliefs without needing to know the true state of an individual respondent.

One might argue that a social desirability bias is less of a problem when eliciting social expectations, because people will be more ready to reveal that *others* are doing or approving of something socially undesirable. This is only true to a certain extent, as people might still be reluctant to admit that their community—their in-group—is doing something undesirable in the eyes of the surveyors. We suspect that this may happen in interviews that take place in small and closely knit communities (usually villages). Even if anonymity is guaranteed, subjects may feel compelled to respond in ways that put their fellow villagers in a positive light.

Yet another major problem in assessing social expectations is that they may not be accurate, in that respondents do not have an incentive to seriously guess what others really approve or disapprove of, and might be induced to project their own preferences and beliefs.

## TOOLS FOR ACCURACY: MONETARY INCENTIVES AND ECONOMIC EXPERIMENTS

Even if a randomized response technique may solve the social desirability problem in assessing truthful personal normative beliefs, we are left with the problem of accuracy in assessing social expectations. A potential solution that should be particularly effective in both experimental and field settings is to incentivize the elicitation of empirical and normative expectations. When accurate responses hold the promise of reward (i.e., when accuracy is incentivized), respondents are motivated to try hard to make an accurate guess (Osband, 1989; see also Goetz et al., 1984). Incentives ensure effortful thinking that can avoid some of the biases to which “automatic” or System 1 thinking is subject (Epley and Gilovich, 2005). Importantly, these incentives for accuracy provide an extra motivation to overcome social desirability and answer honestly (Osband, 1989). If respondents typically want to paint their community in a good light (out of social desirability motivations), monetary incentives will provide adequate reasons to overcome these motivations and respond more accurately. Experiments on public goods games have shown that elicited expectations about other subjects’ contributions are more accurate when the elicitation is incentivized (Gächter and Renner, 2010)^4^. Note, however, that some scholars do not find large differences (Delavande et al., 2011; Eriksson and Strimling, 2014, p. 367).

In what follows, we take a look at how such incentives are used in the economic experiments on fairness norms by Bicchieri and Chavez (2010, 2013)^5^. Their experiments employ a variant of the Ultimatum Game, a game often used in experimental economics. One participant, the “proposer,” received 10 USD and has to propose a division of the money between him/herself and another participant, the “responder.” The proposer has the following three options: (1) $5 for him/herself and $5 for the responder; (2) $8 for him/herself and $2 for the responder; and (3) flip a coin and, if it is heads, it is $5 for him/herself and $5 for the responder or, if it is tails, it is $8 for him/herself and $2 for the responder. Then the responder could either accept or reject the proposal. If the responder accepts, both players receive the amounts proposed. If the responder rejects, nobody receives anything—it is an ultimatum. The possibility of rejection means that the responder can “punish” a proposer for an unfair proposal. But punishment is costly: if a responder rejects an $8-$2 proposal, the opportunity cost is $2.

The experiment was designed to find out whether proposers’ behavior is guided by a fairness norm, as opposed to a generic preference for fairness. The $8-$2 option is clearly unfair. But note that both the $5-$5 option and the coin-flip option could be justified as fair. The $5-$5 option could be perceived as a *fair outcome* because it divides payoffs equally. On the other hand, the coin-flip option could be perceived as a *fair procedure* because it is impartial, although it has an unequal expected payoff of $6.5 versus $3.5. The authors wanted to know whether proposers, torn between self-interest and fairness, would go for the coin flip—following the fairness norm that best serves their interests. They also wanted to know whether players indeed perceived both $5-$5 and the coin flip as fair options and, more importantly, whether players’ normative expectations were mutually consistent, a sign that a social norm exists. Assessing the existence of a social norm is only the first step though. We then have to study under which conditions a norm will be followed, i.e., whether a norm has causal power. In what follows, we discuss all these steps, from the elicitation of behavior and social expectations to the causal influence of social expectations on behavior.

Let us start by summarizing the results on the proposers’ behavior. Bicchieri and Chavez (2013) found that 12% of the proposers chose the selfish option $8-$2, and 88% chose one of the other two options. 44% of the proposers chose the coin flip and the other 44% chose $5-$5. Note that these results give a more reliable picture of people’s fairness behavior than surveys could give. Bicchieri and Chavez’s experiment elicits people’s behavior with real monetary consequences, which reduces the potential social desirability bias that could occur in a survey. If the authors had asked people in a survey what they would hypothetically choose, probably less people would have admitted preferring the selfish option $8-$2—or the self-serving “fair” option of flipping a coin.

Now, if proposers were influenced by social norms, this would mean that their behaviors should depend on, first, their empirical expectations about the behaviors of other proposers and, second, their normative expectations about the personal normative beliefs and the punishing behaviors of the responders. Moreover, if there is indeed a social norm, it is possible that it is sustained by false expectations and that there is pluralistic ignorance, i.e., perceived and objective consensus are inconsistent (Bicchieri, 2006).

Because they were specifically interested in the proposers’ beliefs about fairness, Bicchieri and Chavez (2010; 2013) did not elicit proposers’ empirical expectations. However, they did elicit the responders’ empirical expectations, and the questions they asked responders provide a good example for eliciting proposers’ empirical expectations:

Please guess how many Proposers will choose:

(1) $5 for Proposer and $5 for Responder: _________.

(2) $8 for Proposer and $2 for Responder: _________.

(3) Let a coin flip decide: _________.

For each line in which your guess is correct, you will earn a $1 bonus.

Note how they incentivized the question to increase the reliability of the answers. Systematically incentivizing social-expectations questions was one of the novelties of Bicchieri and Chavez (2010, 2013). Bicchieri and Chavez (2013) found that on average responders thought 44% of the proposers would choose $5-$5, 28% would choose $8-$2, and 28% would choose the coin flip. Since we know what proposers actually chose, we can evaluate the accuracy of the empirical expectations (and pay subjects for correct guesses). See **Table 2** for a comparison of empirical expectations with actual behavior. Additional analyses of the data of Bicchieri and Chavez (2013) shows that empirical expectations were only partly accurate. Responders’ empirical expectations of the proportions of proposers choosing $5-$5 does not significantly differ from the actual proportion (Wilcoxon Sign-rank test, *p* = 0.793). However, responders on average overestimate the proportion of proposers choosing $8-$2 and underestimate the proportion of proposers choosing the coin flip option (Wilcoxon Sign-rank test, *p*-values < 0.01). Though the discrepancy between responders’ empirical expectations about proposers’ behavior and proposers’ actual behavior was not relevant to the questions asked in Bicchieri and Chavez (2010, 2013), they provide a useful example of how such discrepancies could be measured also for proposers’ empirical expectations and how proposers’ behavior may strongly correlate with their expectations of what other proposers do.

**Table 2:** Summary of data on behaviors, personal normative beliefs, rejection and social expectations in Bicchieri and Chavez (2013).

	$5-$5	$8-$2	Coin flip
Behavior of proposers (% of proposers)	44%	12%	44%
Empirical expectations of responders (mean responder beliefs about % of proposers)	44%	28%	28%

Personal normative beliefs of responders (% of responders)	100%	22%	52%
Proposers’ normative expectations about Responders’ personal normative beliefs (mean proposer beliefs about % of responders)	100%	10%	79%

Rejection by responders (% of responders)	0%	25%	0% if $5-$5 0% if $8-$2
Proposers’ expectations of responders’ rejection (% of proposers expecting majority to punish)	0%	52%	0% if $5-$5 22% if $8-$2

Next, let us take a look at how Bicchieri and Chavez (2010; 2013) elicited the proposers’ normative expectations about responders and how we can test the accuracy of these expectations. First the authors elicited the personal normative beliefs of responders by asking the following:

Please mark any options you believe are fair options. You are free to choose none of the options, one, or more than one option. Your answer will not affect your payment.

(1) $5 for Proposer and $5 for Responder [ ].

(2) $8 for Proposer and $2 for Responder [ ].

(3) Let a coin flip decide [ ]^6^.

Then the authors elicited the proposers’ normative expectations about the responders with the following question:

Please guess how many Responders … have selected each of the options in the above question as fair options:

(1) $5 for Proposer and $5 for Responder _________.

(2) $8 for Proposer and $2 for Responder _________.

(3) Let a coin flip decide _________.

For each line on which your guess is correct, you will earn a $1 bonus.

A majority of proposers thought that 100% of responders consider $5-$5 fair, that a large majority of responders consider $8-$2 unfair, and that a large majority of responders think the coin flip is fair. This tells us that proposers’ normative expectations were mutually consistent, i.e., there was agreement that a fairness norm exists and applies to their situation. The authors found that the choices of the proposers correlate significantly and strongly with their normative expectations. For instance, proposers were more likely to choose the coin flip the more responders they thought would deem the coin flip fair. So proposers were plausibly influenced by a social norm of fairness.

To know whether the proposers’ normative expectations about the responders were accurate, they must be compared with the personal normative beliefs of the responders. As we mentioned, the authors found that 100% of the responders thought $5-$5 was fair, 22% thought $8-$2 was fair, 52% thought the coin flip was fair (see also **Table 2**). Additional analyses confirm that normative expectations overestimated the perceived fairness of the coin flip and underestimated the acceptability of $8-$2, since normative expectations significantly differ from the actual normative beliefs of responders (Wilcoxon Sign-rank test, *p*-values < 0.01 for both $8-$2 and coin flip)^7^.

Finally, Bicchieri and Chavez (2010) also elicited proposers’ expectations about the behaviors of the responders, that is, their tendency to reject certain proposals. They asked proposers the following questions:

• Will the majority of Responders accept $5-$5 not resulting from a coin flip? _________.
• Will the majority of Responders accept $8-$2 not resulting from a coin flip? _________.
• Will the majority of Responders accept $5-$5 resulting from a coin flip? _________.
• Will the majority of Responders accept $8-$2 resulting from a coin flip? _________
    For each item you answer correctly, you will earn a $0.5 bonus.

No proposers expected the majority of responders to reject when the proposal was $5-$5—whether this was the chosen option or the outcome of the coin flip. However, 52% of the proposers expected the majority to reject when $8-$2 was chosen, and 22% expected the majority to reject when the coin flip was chosen and the outcome was $8-$2. The authors showed that the proposers’ behavior correlates with their rejections expectations. Again, the accuracy of the expectations about rejection can be tested by comparing them with actual rejecting behavior. As it turns out, only 25% of the responders rejected $8-$2 proposals, while nobody rejected coin-flip proposals—not even when the outcome turned out to be $8-$2 (see **Table 2**). Of course nobody rejected $5-$5 proposals. Hence, expectations about rejections overestimated the amount of rejections both for the $8-$2 and the coin flip proposals.

We have shown that the Bicchieri and Chavez (2010, 2013) experiments were able to find out to which extent people’s behavior depended on their empirical and normative expectations. However, the fact that behavior correlates with social expectations does not mean that a change in social expectations will necessarily *cause* a change in behavior. In other words, knowing that a social norm exists is just a first step: we have to know under which conditions it will be followed. Since conditional preferences are necessary to follow a norm, and preferences are conditional on social expectations, we have to check whether *manipulating* social expectations will cause a change in behavior. For organizations designing programs to change harmful practices, it is useful to know whether changing social expectations will actually change behavior.

Earlier, we discussed the problem of finding out the causal role of social expectations (and other potential motives) in the context of surveys. Now, experiments are an excellent tool to discover causal relationships, and in another experiment Bicchieri and Xiao (2009) did exactly that. Their experiment employed a variant of the Dictator Game, another game often used in experimental economics, similar to the Ultimatum Game. One participant, the “dictator,” who is called the “divider” in the experiment, received 10 USD and had to divide the money between him/herself and another participant, the “receiver.” The divider had the following seven options: $9-$1 ($9 for him/herself and $1 for the receiver), $8-$2, $6-$4, $5-$5, $4-$6, $8-$2, and $9-$1. Unlike in the Ultimatum Game, the receiver cannot reject the offer.

Bicchieri and Xiao (2009) manipulated social expectations by giving dividers some (true) information about what other dividers had said or done in previous experiments with the same Dictator Game. They then asked dividers about their expectations about the behavior and beliefs of other dividers in the present game. The question was whether information about previous games would influence present expectations and behavior. With regard to empirical expectations, some dividers were manipulated to expect *fair* behavior from other dividers, while other dividers were manipulated to expect *selfish* behavior. Similarly, with regard to normative expectations, some dividers were manipulated to expect others to believe that one should be *fair*, while other dividers were manipulated to expect others to believe that one should be *selfish*. **Table 3** shows the messages that manipulated social expectations.

**Table 3:** Messages used in Bicchieri and Xiao (2009) to manipulate social expectations.

	Empirical expectations	Normative expectations
Fair	“60% of the dividers who participated in a session of this experiment last year **shared the amount approximately equally (i.e., chose option $5-$5 or $6-$4)**.”	“60% of the dividers who participated in a session of this experiment last year **said that dividers should share the amount approximately equally (i.e., choose option $5-$5 or $6-$4)**.”
Selfish	“60% of the dividers who participated in a session of this experiment last year **approximately maximized their own earnings (i.e., chose option $9-$1 or $8-$2)**.”	“60% of the dividers who participated in a session of this experiment last year **said that dividers should approximately maximize their own earnings (i.e., choose option $9-$1 or $8-$2)**.”

*The part of the message that differs is in bold.*

As a manipulation check, the authors elicited the dividers’ social expectations, in an incentivized way, like in Bicchieri and Chavez (2010, 2013). The information given strongly influenced players’ social expectations. More important, the expectation manipulations influenced dividers’ behavior. For instance, a regression analysis showed that the probability that a dictator chooses a fair option ($5-$5 or $6-$4) increases by about 6% if his/her empirical expectations of fair choices changes from 45 to 50%. The Bicchieri and Xiao (2009) experiment allows one to conclude that social expectations have causal influence on fair behavior.

## MORE REALISTIC TOOLS: IN-KIND INCENTIVES AND VIGNETTES

The economic experiments discussed above should be considered as ideal tools rather than exact molds. When studying *norms in the wild*, experiments like the ones just discussed are likely to be problematic. Nevertheless, understanding such experiments is useful. By understanding the ideal, one can start thinking about alternatives that can achieve similar results. For example, experiments tell us that incentivizing answers about social expectations solves the accuracy problem, a tool we may use also in surveys. Experiments also let us measure both consensus and compliance. If normative expectations are mutually consistent we can be reasonably sure that a social norm exists, and manipulating social expectations tells us if the norm has causal power, i.e., under which conditions it will affect behavior. Modified surveys can tell us if normative expectations are mutually consistent, but they may not work if the goal is to measure causal efficacy. In this section, we discuss a few alternatives, like in-kind incentives and vignettes.

When studying child marriage, it is not an option to “manipulate” expectations and then observe whether behavior changes. We cannot invite fathers to the lab and ask them to make real decisions about marrying off their daughters. The best we can do is ask them at what age they married off—or will or would marry off—their daughters. But we can still incentivize the elicitation of social expectations. For deontological reasons, organizations might be weary of using monetary incentives. However, one could reward correct answers with useful presents, like food or vouchers. Below is an example of incentivized questions about people’s social expectations regarding child marriage:

• EMPIRICAL EXPECTATION: “In what follows you will be asked to make a guess. If you guess correctly, you win 1 pound of dried figs. We interviewed many men (at least 40 years old) with married daughters in your community, and we asked them the following question: *At what age did your daughter(s) get married?* Out of 100 married girls/women, how many do you think got married before they were 18 years old?”
• NORMATIVE EXPECTATION: “In what follows you will be asked to make a guess. If you guess correctly, you win 1 pound of dried figs. We interviewed many men (at least 40 years old) with married daughters in your community, and we asked them the following question: *Some girls get married before they are 18 years old. Is this good?* Out of 100 men, how many do you think answered that it is good?”

Asking such questions enables one to elicit social expectations and evaluate their accuracies in ways similar to the experimental methods used in Bicchieri and Chavez (2010, 2013), and Bicchieri and Xiao (2009). Such questions require careful design. In particular, if we want to be able to evaluate the accuracy of the social expectations of respondents, we have to make sure that empirical-expectation questions match behavior questions, and normative-expectation questions match personal-normative-belief questions. Suppose we asked people about their personal normative beliefs with the question suggested earlier (“Some girls get married before they are 18 years old. Is this good?”), but, instead of the normative-expectation question suggested above, we ask the following modified (and ill-matched) version of the normative-expectation question: “Do you think people in your community believe that it is a father’s duty to marry off daughters as soon as possible?” Because this question does not match the personal-normative-belief question, it becomes impossible to evaluate the accuracy of the normative expectations.

One might wonder whether manipulating social expectations raises deontological concerns. First, note that the Bicchieri and Xiao (2009) experiment did not involve deception, which would violate a fundamental methodological precept of experimental economics. Both the messages stressing fairness and the messages stressing selfishness were true, since there had been actual sessions with these particular outcomes. Expectations were manipulated merely by being selective in presenting information. Still, organizations might find experiments with manipulations difficult to implement.

If experiments with manipulations are difficult in the field, surveys that would have to ask hypothetical questions do not fare much better. Imagine asking a father the hypothetical question what he would do *if* he were to realize that most people in his reference network have decided to abandon child marriage or have become strongly opposed to the practice. A likely answer from the father would be that this is not and probably will never be occurring, as he *knows* what people in his network do and believe. Hypothetical questions are difficult, as they require the capability to answer “what if” questions, and imagine scenarios that may seem prima facie impossible. Contrary-to-fact hypotheticals, for example, require the ability to assume as true a claim that conflicts with what is accepted as true, and the lack of such ability may lead to deny that the suggested scenario is possible. It may be easier to answer hypothetical questions about fictitious characters than questions about actual family and friends. This is what vignettes accomplish.

Similar to experiments, vignettes make use of manipulation to arrive at causal knowledge^8^. Vignettes tell short stories about imaginary characters in specific scenarios (Alexander and Becker, 1978; Finch, 1987). Asking respondents about these stories can effectively elicit beliefs and expectations: they are particularly useful when the questions being asked are socially sensitive and subject to social desirability biases (Finch, 1987). In these stories, respondents will not feel the same obligatory pressures to respond in a particular way. These hypothetical scenarios provide an unthreatening and impersonal avenue for exploring respondents’ attitudes or beliefs about a sensitive topic.

For instance, we can give respondents stories about a father in some community who is wondering whether to marry off his young daughter or to let her finish school. We can then ask respondents what they think this father would ultimately do, why he would do that, and whether it is what the father should do (see the example questions in **Table 4**). We can also vary what others in this fictional father’s community are doing or what they are approving and disapproving of (i.e., we can manipulate the social expectations of the protagonist of the story). In the stories some respondents get, all the other men are arranging early marriages, whereas in alternative stories the other men send their daughters to school until they finish high school. In some stories, all the other men think that marriage and chastity is infinitely more important than education for girls, whereas in other stories the other men in the fictitious community recognize the importance of education (see **Table 4**).

**Table 4:** Vignettes that manipulate social expectations.

	Empirical expectations	Normative expectations
Marry early	“Mr. Badji has been visited by a very respectable family who want their son to marry Mr. Badji’s daughter. It is a very good opportunity, but Mr. Badji’s daughter is 15 years old and still going to school. **Most girls in the village marry before they are 16 years old**.”	“Mr. Badji has been visited by a very respectable family who want their son to marry Mr. Badji’s daughter. It is a very good opportunity, but Mr. Badji’s daughter is 15 years old and still going to school. **People in the village say a good father arranges a good marriage as soon as a good opportunity arises**.”
Marry late	“Mr. Badji has been visited by a very respectable family who want their son to marry Mr. Badji’s daughter. It is a very good opportunity, but Mr. Badji’s daughter is 15 years old and still going to school. **Most girls in the village marry after finishing high school, at 18 years old or later**.”	“Mr. Badji has been visited by a very respectable family who want their son to marry Mr. Badji’s daughter. It is a very good opportunity, but Mr. Badji’s daughter is 15 years old and still going to school. **People in the village say a good father arranges a good education first, and only after that he arranges a good marriage**.”

*The part of the story that differs is in bold. Questions to be asked about these vignettes are: In your opinion, will Mr. Badji ultimately agree to the marriage of his daughter [behavior]? Why [preferences]? What (if anything) might drive Mr. Badji to agree to the marriage [preferences]? What (if anything) might drive Mr. Badji to say no to the marriage [preferences]? Do you think Mr. Badji should agree to the marriage [personal normative belief]?*

Another important variable to manipulate is the role in the community that people depicted in the story have. In some stories, it might be *other men* in the village that say that good fathers arrange marriages as soon as possible. In other stories, it might be the *mother in law*, or *religious and community leaders*. By learning exactly whose opinions and behaviors matter, we can identify what we earlier called the reference network. Identifying the reference network is useful because, if we can change the opinions and behaviors of this reference network, we are more likely to change the opinions and behaviors of the entire community.

Note that in vignettes like the ones we suggested we would be manipulating the social expectations of the protagonist of the story, and not those of the respondent. However, most individuals are subject to what is known as a “false consensus effect” in that, when not aware of dispositional or person-specific information, they infer that a decision maker would behave as if they themselves would when in a particular situation (Ross et al., 1977; Mullen et al., 1985). When respondents think about what the protagonist would do, they imagine what they would do if they were in the position of the protagonist. Thus, vignettes indirectly teach us something about how the respondent would react. Similarly, by manipulating the protagonist’s social expectations, it is as if we were manipulating the respondent’s social expectations. In that sense, vignettes are quasi-experiments. Moreover, the indirectness of vignettes helps eliminate the social desirability bias, since we ask people what they think some other individual in another community will think and do instead of posing the questions directly about themselves.

By manipulating social expectations, we will not only be able to say whether something is a social norm or not, but we will also be able to spot individual differences in sensitivity to social norms. In other words, we will find out who is more *autonomous* than others. Autonomy is the ability and desire to make one’s own choices, to choose what one really wants, and to reflect on what one might want (Chirkov et al., 2011; Bavetta and Navarra, 2012, Chap. 3), rather than having others make choices for you and decide for you what you want. Many child marriage programs aim at empowering women (e.g., Sood et al., 2007)—at increasing their level of autonomy—so that they can abandon harmful social norms and perhaps even take the lead in the collective process of change. Hence, measuring individual differences in autonomy and norm sensitivity can add substantial value to the design of effective interventions as well as child marriage M&E. To gain a comprehensive understanding of respondents and their motivations, it would be useful to analyze vignette data in conjunction with survey data.

Despite the many benefits that the use of vignettes offers, it is worth acknowledging they do have some potential pitfalls. Any vignette designer must be careful that the vignette is not so complex or alien that the respondent has trouble understanding or relating to it (Finch, 1987). Moreover, it is possible that respondents will “fill in” the missing information of a particular vignette in different ways; for example, some respondents might imagine that Mr. Badji is Muslim, and others might imagine that he is Hindu (or Christian, or agnostic). Thus, it is worth making any critical details explicitly stated so that respondents do not imagine the scenarios in different lights (thereby adding undesirable noise to the data)^9^.

## CONCLUSION

In this paper, we presented a theoretical framework that can guide the M&E of progress in ending child marriage. M&E is difficult because there are many possible explanations of child marriage. We have shown how a general framework can be built on the basis of a simple model that explains the behavior of individuals in terms of the *preferences* they have, the *options* they face and the *beliefs* they hold about these options. We have gradually refined this model by introducing different kinds of beliefs—non-social and social—and different kinds of preferences—unconditional and conditional. Child marriage has proven difficult to curb, and one of the reasons is undoubtedly the fact that it is sustained by preferences conditional on expectations about others’ behaviors and beliefs.

There are empirical, precise tools we can use to assess social expectations, to diagnose collective practices, and ultimately to guide interventions and M&E. We have discussed here surveys, experiments, and vignettes. These tools have been commonly used in social-science empirical research. To be adapted to a general explanation of child marriage they need several modifications. Surveys should be incentivized and enriched by asking clear social-expectations questions. Experiments can teach us how to uncover social norms and how to manipulate expectations to understand what induces people to obey them. Vignettes are useful in the field, where it is impossible to manipulate social expectations directly. They allow for indirect assessment of how changes in social expectations would affect behavior, and whether some expectations are more important than others. By guiding empirical research with precise theoretical tools we can accomplish what has been difficult, if not impossible, to do up to know: to provide a general explanation for some common practice that has often defied the best-intentioned intervention.

## Conflict of Interest Statement

The authors declare that the research was conducted in the absence of any commercial or financial relationships that could be construed as a potential conflict of interest.

## Footnotes

1 Child marriage is a marriage in which at least one of the spouses is less than 18 years old. In most cases, it is the girl who is still a child. Child marriage is also more problematic for girls. Therefore, we focus on girls in this paper, although for simplicity we will still talk about child marriage.

2 For instance, in their interesting review of programs to end child marriage, Malhotra et al. (2011) write: “efforts …attempt to change underlying *social norms* that perpetuate the *practice* of child marriage” (p. 6, emphasis added); “parental education and community mobilization attempt to change *social norms* and forge a more supportive, less punitive environment for girls and families who are willing and ready to change the *custom* of early marriage” (p. 13, emphasis added); and “child marriage prevention programs …aim to change deeply entrenched *social and cultural* practices” (p. 24, emphasis added). But what is the difference between a “practice,” a “custom,” and a “social norm,” and what does it mean for a practice to be “social” or “cultural”?

3 We cannot say of personal normative beliefs that they are false, because these beliefs do not refer to facts or people that we can at least in principle observe. Factual beliefs and social expectations, however, can clearly be false.

4 A potential problem is that Gächter and Renner (2010) also found that eliciting beliefs (1) increases contributions and (2) makes the level of contributions depend to a greater extent on beliefs. However, Bicchieri and Chavez (2010) ran one treatment in which beliefs were elicited *before* playing the Ultimatum Game and another treatment in which beliefs were elicited only *after* playing the game—so that the elicitation could not influence behavior. Like Gächter and Renner (2010), they found that eliciting beliefs before the game has an influence on behavior in the game. This is an argument for eliciting beliefs after the game, which is what Bicchieri and Chavez (2013) did, and it is these data that we will use in what follows.

5 Note that we will only discuss the parts of the experimental design and those results that are relevant to our methodological discussion. Their experiment had three information treatments, but we discuss only one. Moreover, we do not discuss any of the author’s findings on norm evasion and norm manipulation which are key to their 2013 paper but are not so relevant here. Finally, we only use the data of Bicchieri and Chavez (2013).

6 Note that it is impossible to reward subjects with a $1 bonus for accurately stating their own fairness beliefs, because the only way to check the accuracy is to ask these very subjects. Personal normative beliefs cannot be incentivized that way. But there are other ways, beside the randomized response method we mentioned earlier: one can for instance actually implement decisions about what is the fairest distribution between two other unrelated subjects (Bernard et al., 2012).

7 It is interesting to notice that, the normative expectations of responders (about other responders) were similar to the proposers’ normative expectations as they also underestimated the proportion of responders who view $8-$2 as fair (mean = 15 vs. 22%, Wilcoxon Sign-rank test, *p* = 0028). However, there is no evidence that they overestimated the proportion of responders who view the coin flip as fair (mean = 46 vs. 52%, *p* = 0.223).

8 While KAP-style surveys are still the norm in social-norms research, some scholars are already using vignettes to assess whether a social norm is present (e.g., Sorenson and Taylor, 2005; Mathew and Boyd, 2011; for other examples see Strimling and Eriksson, 2014).

9 Interestingly, Finch (1987) discusses how some ambiguity in a vignette can be a positive feature, as it grants respondents the ability to interpret the situation in their own terms.
